# Maize/soybean strip intercropping produces higher crop yields and saves water under semi-arid conditions

**DOI:** 10.3389/fpls.2022.1006720

**Published:** 2022-11-01

**Authors:** Muhammad Ali Raza, Hassan Shehryar Yasin, Hina Gul, Ruijun Qin, Atta Mohi Ud Din, Muhammad Hayder Bin Khalid, Sajad Hussain, Harun Gitari, Amjed Saeed, Jun Wang, Esmaeil Rezaei-Chiyaneh, Ayman El Sabagh, Amir Manzoor, Akash Fatima, Shakeel Ahmad, Feng Yang, Milan Skalicky, Wenyu Yang

**Affiliations:** ^1^National Research Center of Intercropping, The Islamia University of Bahawalpur, Bahawalpur, Pakistan; ^2^College of Agronomy, Sichuan Agricultural University, Chengdu, China; ^3^Gansu Academy of Agricultural Sciences, Lanzhou, Gansu, China; ^4^National Center for Industrial Biotechnology, Pir Mehar Ali Shah-Arid Agricultural University, Rawalpindi, Pakistan; ^5^Hermiston Agricultural Research and Extension Center, Oregon State University, Hermiston, OR, United States; ^6^College of Agriculture, Nanjing Agricultural University, Nanjing, China; ^7^Department of Agricultural Science and Technology, School of Agriculture and Enterprise Development, Kenyatta University, Nairobi, Kenya; ^8^Shaanxi Key Laboratory of Earth Surface System and Environmental Carrying Capacity, College of Urban and Environmental Science, Northwest University, Xian, China; ^9^Department of Plant Production and Genetics, Faculty of Agriculture, Urmia University, Urmia, Iran; ^10^Department of Field Crops, Faculty of Agriculture, Siirt University, Siirt, Turkey; ^11^Institute of Plant Breeding and Biotechnology, Muhammad Nawaz Sharif-University of Agriculture, Multan, Pakistan; ^12^Department of Agronomy, Bahauddin Zakariya University, Multan, Multan, Pakistan; ^13^Department of Botany and Plant Physiology, Faculty of Agrobiology, Food and Natural Resources, Czech University of Life Sciences Prague, Prague, Czechia

**Keywords:** land productivity, water use efficiency, competition, sustainability, economic profit

## Abstract

Sustainable increases in crop production require efficient use of resources, and intercropping can improve water use efficiency and land productivity at reduced inputs. Thus, in a three-year field experiment, the performance of maize/soybean strip intercropping system differing with maize plant density (6 maize plants m-2, low, D1; 8 maize plants m-2, medium, D2; and 10 maize plants m-2, high, D3) was evaluated in comparison with sole maize or soybean cropping system. Results revealed that among all intercropping treatments, D2 had a significantly higher total leaf area index (maize LAI + soybean LAI; 8.2), total dry matter production (maize dry matter + soybean dry matter; 361.5 g plant-1), and total grain yield (maize grain yield + soybean grain yield; 10122.5 kg ha-1) than D1 and D3, and also higher than sole maize (4.8, 338.7 g plant-1, and 9553.7 kg ha-1) and sole soybean (4.6, 64.8 g plant-1, and 1559.5 kg ha-1). The intercropped maize was more efficient in utilizing the radiation and water, with a radiation use efficiency of 3.5, 5.2, and 4.3 g MJ-1 and water use efficiency of 14.3, 16.2, and 13.3 kg ha-1 mm-1, while that of intercropped soybean was 2.5, 2.1, and 1.8 g MJ-1 and 2.1, 1.9, and 1.5 kg ha-1 mm-1 in D1, D2, and D3, respectively. In intercropping, the land and water equivalent ratios ranged from 1.22 to 1.55, demonstrating that it is a sustainable strategy to improve land and water use efficiencies; this maximization is likely associated with the species complementarities for radiation, water, and land in time and space, which resulted in part from competition avoidance responses that maximize the economic profit (e. g., 1300 US $ ha-1 in D2) over sole maize (798 US $ ha-1) or sole soybean (703 US $ ha-1). Overall, these results indicate that optimizing strip intercropping systems can save 20–50% of water and land, especially under the present scenario of limited resources and climate change. However, further research is required to fully understand the resource capture mechanisms of intercrops in intercropping.

## Introduction

Food security is a prerequisite for ensuring national security and human survival. The global human population is projected to cross nine billion in 2050 (Thornton et al., 2014). Thus, to fulfill the enhanced demands of an increasing population for food and feed, it is estimated that the current crop yield needs to be increased by 50% in 2030 and 100% in 2050 (Li et al., 2020). The continuous decline in cultivable lands due to urbanization and industrialization has limited the further expansion in cultivation area of cereals (e. g., maize; *Zea mays* L.) and legumes (e. g., soybean; *Glycine max* L.). This situation is more serious in the developing countries (e. g., China, Pakistan, and India) that have more population and less cultivable land (Du et al., 2017; Iqbal et al., 2018). Furthermore, researchers have reported that the expansion in the cultivation area for food crops is the leading cause of deforestation in many regions that adversely affect the environment (Barona et al., 2010). Therefore, in the present scenario of limited resources (i. e., land and water) and climate change, it is important to develop new cropping systems (i. e., intercropping or agroforestry), which can increase crop yields by effectively using the limited resources without affecting the environment.

Intercropping, the cultivation of two or more crop species on the same land, provides opportunities for sustainable crop production and agricultural intensification (Feng et al., 2019). Intercropping results in higher crop yield at the system level (grain yield of species one + grain yield of species two) and less yield variation than mono-cropping systems (Martin-Guay et al., 2018). This higher and stable yield, particularly with reduced inputs, are mainly ascribed to resources (i. e., water, sunlight, and nutrients) complementarity (Liu et al., 2017; Gitari et al., 2018; Raza et al., 2019), in which intercrop species utilize available resources more adequately due to different spatial (Raza et al., 2021a), temporal (Yang et al., 2017), and phenological characteristics (Li et al., 2013). The intra- and interspecific competition (Yang et al., 2015), availability of environmental resources (Liu et al., 2017), and planting density of the intercrop species influenced the degree of resource complementarity (Ren et al., 2016) and the yield of intercropping (Hauggaard-Nielsen and Jensen, 2005). For instance, maize and soybean produced larger relative grain yields in strip intercropping than in mono-cropping (Chen et al., 2017; Du et al., 2017); and intercropping of maize with soybean achieved high land productivity (estimated as a land equivalent ratio; LER) with high maize planting density compared to low maize planting density under strip intercropping (Muoneke et al., 2007). These findings conclude that strip intercropping produces higher yields at the system level than mono-cropping due to complementarity and facilitation interactions.

Determining the optimum planting density of intercrop species is a paramount for higher crop yields in intercropping. Compared with mono-cropping, crops in intercropping use planting space more efficiently and effectively (Raza et al., 2020). The optimum planting density in intercropping outweighs the optimum planting density in mono-cropping (Willey and Osiru, 1972). Nevertheless, the optimum planting density of one intercrop species at one location, i. e., maize in maize/soybean intercropping at Sichuan under high-rainfall conditions (Feng et al., 2020), maize in maize/wheat intercropping at Wageningen under medium-rainfall conditions (Gou et al., 2016), maize in maize/pea intercropping at Gansu under low-rainfall conditions (Mao et al., 2012), and maize in maize/pigeon pea intercropping at Trinidad under irrigated conditions (Dalal, 1974), may not be applicable to other sites because of the regional variations in soil properties (water holding capacity, total available nitrogen, phosphorus, and potassium, and organic matter) and weather (precipitation, temperature, and solar radiation). However, lack of appropriate study and relevant literatures on determining the optimum planting density of maize in cereal/legume intercropping systems under irrigated conditions, especially in semi-arid areas (high-temperature regions, where farmers are using extra water for the production of cereals and legumes).

Researchers have previously reported that a higher planting density of intercropped maize resulted in greater intercropping advantages (Willey and Osiru, 1972; Muoneke et al., 2007). Whereas it significantly affects the competitive interactions between intercrops; for instance, the dominance of maize over soybean was enhanced with increased maize density, which ultimately decreased the grain yield of soybean in maize/soybean intercropping (Muoneke et al., 2007). In addition, the planting density of intercrop species, especially of tall crops, adversely affects the root growth and distribution (Hauggaard-Nielsen et al., 2001), sunlight transmittance (Li et al., 2001), leaf area development (Prasad and Brook, 2005), dry matter production (Ren et al., 2016), and resource capturing (Gao et al., 2009) of understory crops in cereal/legume intercropping systems. However, most past studies on the plant density response of intercrops have mainly been conducted by changing the row ratio or sowing proportions (Ofori and Stern, 1987; Ijoyah and Fanen, 2012; Mao et al., 2012). Thus, the response of intercrops to equal row-ratio and sowing proportion under strip intercropping systems remains unclear. The interaction (below and above ground) of intercrops species has been reported to enhance the water and light utilization efficiency. Furthermore, it has been rarely investigated how changing maize planting density affects the interspecific interactions, competition for the acquisition of available resources (i. e., water and radiation), and land productivity of maize/soybean strip intercropping (maize/soybean intercropping) under irrigated conditions. Therefore, the main aims of this study were to determine the effects of changing maize planting density on (i) growth and crop yields of maize and soybean in maize/soybean intercropping, (ii) resource (water or sunlight) utilization dynamics of intercrops under maize/soybean intercropping, and (iii) land productivity and economic viability of maize/soybean intercropping compared to sole cropping of maize and soybean using data from a three-year field experiment.

## Materials and methods

### Field experiments

**Figure 1:**
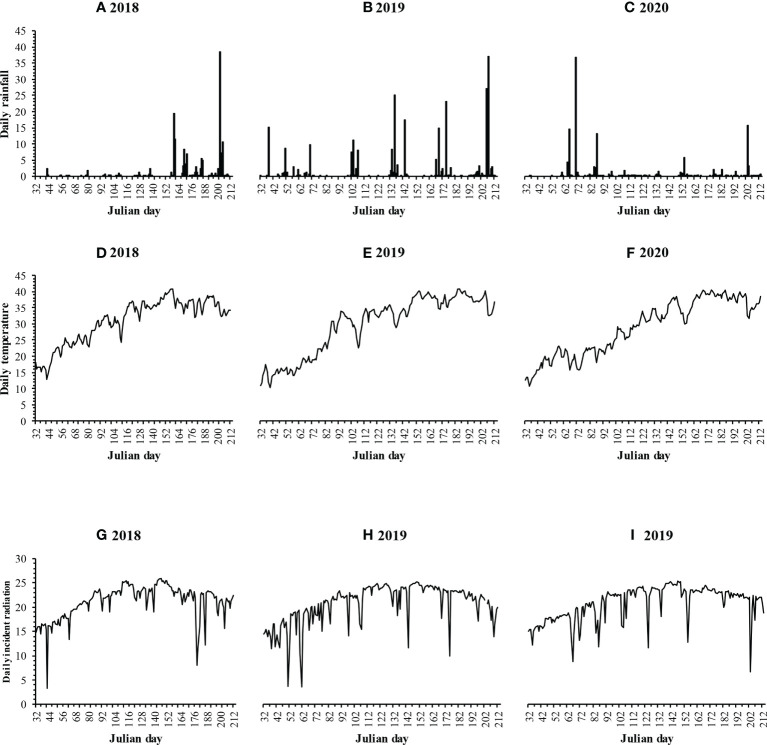
Daily rainfall (mm), temperature (°C), and incident radiation (MJ m^-2^ day^-1^) during the summer season of maize and soybean in 2018, 2019, and 2020.

The field study was conducted in 2018, 2019, and 2020 at Khairpur Tamewali (29.57°N, 72.25°E; altitude 130 m), Bahawalpur, Punjab Province, Pakistan, a research site of Sichuan Agricultural University, P. R. China. The research site has a continental monsoon climate, with a mean annual precipitation of 143 mm and a temperature of 25.7°C. The soil was a sandy clay loam, with 7.7 pH, 7.3 g kg^-1^ organic matter, 0.5 g kg^-1^ total nitrogen (N), 5.0 mg kg^-1^ available phosphorus (P), 341.5 mg kg^-1^ available potassium (K), and 1.47 Mg m^-3^ bulk density. Daily incident solar radiation, air temperature, and rainfall of 2018, 2019, and 2020 are shown in **Figure 1**. During the planting period (from sowing to harvest), total rainfall was 77, 105, and 280 mm in 2018, 2019, and 2020, respectively.

**Figure 2:**
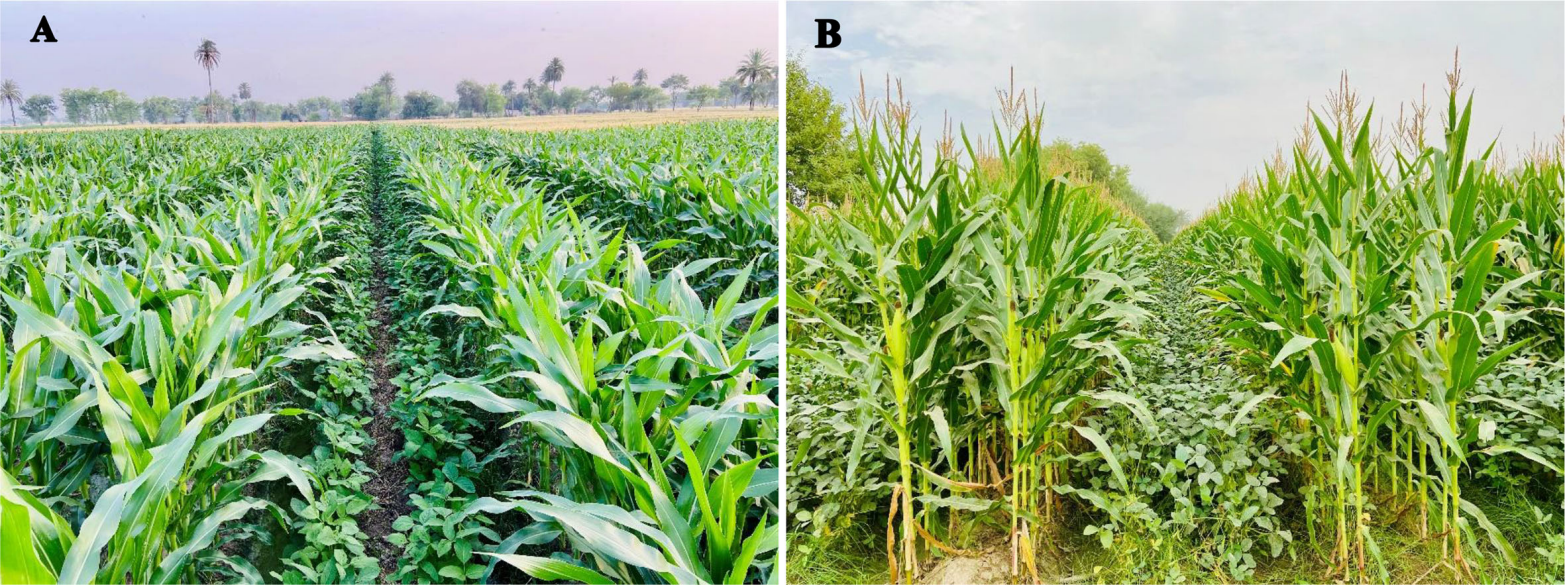
Field demonstration of maize/soybean strip intercropping system. **(A)** Intercrops were at the vegetative growth stage, and **(B)** Intercrops were at the reproductive growth stage (Photos: Muhammad Ali Raza). Location: Punjab Province, Pakistan.

The experiment was laid out in a randomized complete block design with three replications. The study consisted of three maize/soybean intercropping treatments differing with maize plant density (6 maize plants m^-2^, low, D_1_; 8 maize plants m^-2^, medium, D_2_; and 10 maize plants m^-2^, high, D_3_) and two sole cropping treatments of maize (M) and soybean (S). The intercropping treatments comprised of two rows of maize with two rows of soybean in each intercropping strip (**Figure 2**); six intercropping strips were arranged in each intercropping plot. The size of each plot was 144 m^2^ (12 m in width and 12 m in length). The plant configuration (i. e., row spacings, plant distances, and planting densities) in D_1_, D_2_, D_3_, M, and S are presented in **Table 1**. According to the local recommended planting densities, both sole crops were planted: 80000 plants ha^-1^ for maize and 140000 plants ha^-1^ for soybean. In addition, all agronomic practices, i. e., sowing, weeding, and harvesting, were done manually.

**Table 1:** The plant to plant, row to row, strip to strip distances for maize and soybean, and total planting densities of maize and soybean in intercropping and sole cropping systems.

Treatments	Plant distance		Row distance		Strip distance ^**^	Strips/Rows		Total planting density		
	(cm)		(cm)		(cm)	(plot^-1^)		(plants ha^-1^)		
	Maize	Soybean	Maize	Soybean		Maize	Soybean	Maize	Soybean	Total
D_1_*	16.7	7.2	40	40	60	06 ^***^	06	60000	140000	200000
**D_2_**	12.5	7.2	40	40	60	06	06	80000	140000	220000
**D_3_**	10.0	7.2	40	40	60	06	06	100000	140000	240000
**M**	16.7	–	75	–	–	16	–	80000	–	80000
**S**	–	14.3	–	50	–	–	24	–	140000	140000

^*^ The D_1_ (6 maize plants m^-2^, low, D_1_), D_2_ (8 maize plants m^-2^, medium, D_2_), and D_3_ (10 maize plants m^-2^, high, D_3_) represent the three maize/soybean intercropping treatments differing with maize plant density. The M refers to the sole cropping system of maize, and the S refers to the sole cropping system of soybean.

^**^ Strip distance between the strips of maize and soybean in maize/soybean strip intercropping system.

^***^ Each strip of maize or soybean in the maize/soybean strip intercropping system contained two rows of maize or two rows of soybean.

The soybean (determinate) variety ‘NARC-16’ and maize (semi-compact) variety ‘DK-6317’ were used in the study. Both crops were planted and harvested on the same date, on February 03^rd^ in 2018, February 05^th^ in 2019, and February 7^th^ in 2020; and harvested on June 30^th^ in 2018, July 7^th^ in 2019, and July 5^th^ in 2020. Before sowing, for maize, basal N at 120 kg ha^−1^ as urea, P at 205 kg ha^−1^ as diammonium phosphate (DAP), and K at 150 kg ha^−1^ as potassium sulfate (SOP) were applied between maize rows in D_1_, D_2_, D_3_, and M. For soybean, basal N at 75 kg ha^−1^ as urea, P at 150 kg ha^−1^ as DAP, and K at 100 kg ha^−1^ as SOP were used between soybean rows in D_1_, D_2_, D_3_, and S. At the V_6_ and tasseling stages of maize, the second and third doses of N were applied at 60 and 100 kg ha^−1^, respectively, as urea between maize rows under D_1_, D_2_, D_3_, and M. Besides, all treatments were irrigated with the same amount of water across the whole experiment, and the detailed information is shown in **Table 2**. According to the local water application advisory for maize and soybean production, irrigation water was applied, which is equal to 550 ± 100 mm water for both crops depending on the crop or weather conditions. Groundwater was pumped out using a tube well and applied *via* the furrow irrigation method.

**Table 2:** Rainfall (mm), irrigation water (mm), and total water use (mm) of maize and soybean under sole and intercropping systems at the experimental site of Sichuan Agricultural University, Bahawalpur, South Punjab, Pakistan.

Years	Rainfall					Irrigation water ^*^					Total water use (rainfall + irrigation) ^**^				
	Feb	Mar	April	May	June	Feb	Mar	April	May	June	Feb	Mar	April	May	June
**2018**	03	03	04	05	62	60	81	121	121	30	63	84	125	126	92
**2019**	17	09	18	33	28	40	81	121	91	60	57	90	139	124	88
**2020**	01	216	18	14	31	60	00	101	121	50	61	216	119	135	81

^*^ All treatments were irrigated with the same amount of irrigation water by differentiating the treatments.

^**^ During the whole cropping season, the total water use by maize or soybean under sole or intercropping systems was 490 mm in 2018, 498 mm in 2019, and 613 mm in 2020.

### Measurements

Leaf area of maize and soybean was measured five times at 45, 65, 85, 105, and 125 days after sowing (DAS) in all years of this study. For this purpose, three maize and five soybean plants were destructively sampled from each plot at each sampling time. The leaf area of all leaves was determined by multiplying the greatest leaf width and length with the crop-specific co-efficient factor of 0.70 for maize and 0.75 for soybean (Gao et al., 2009). Then, the leaf area index (LAI) was calculated using the following equation (Montgomery, 1911).


$$LAI=\;\frac{\left(Leaf\;area\;plant^{-1}\times Plant\;number\;plot^{-1}\right)}{Plot\;area}$$

Three maize and five soybean plants from each plot were collected at 45, 65, 85, 105, and 125 DAS for total dry matter production and partitioning analysis. Then, all samples were divided into various plant parts (root, straw (leaves + stem + non-grain parts), and grain) and sun-dried for the next seven to ten days to achieve a constant weight and presented as g plant^-1^. The total dry matter (TDM; g plant^-1^) of maize and soybean was determined from the summation of the dry matter of root, straw, and grain. Additionally, the total dry matter (g plant^-1^) of intercropping treatments was calculated from the summation of the total dry matter of maize and soybean in D_1_, D_2_, and D_3_.

To determine the grain yield of maize and soybean, 24 maize-ears and 40 soybean plants were collected from each plot of D_1_, D_2_, D_3_, M, and S at the maturity of both crops. These samples were used to quantify the yield response of maize and soybean to changing planting density in intercropping. All the harvested samples were sun-dried for the next seven to ten days. Then, the dried samples were manually threshed and weighed to determine the maize and soybean grain yield and converted into kg ha^-1^. Additionally, the total grain yield of intercropping treatments was calculated from the summation of the grain yield of maize and soybean in D_1_, D_2_, and D_3_.

To calculate the radiation use efficiency of both crops under different treatments, we first determine the daily total incident solar radiation (MJ m^-2^ day^-1^) using the following equation (Angstrom, 1924).


$$SR=SR_{0\;}\left(a+b\times n/N\right)$$

Where, *SR_0_* was the extraterrestrial radiation. The *a* and *b* were the constants and used for those areas where the data for *SR* is not available (Allen et al., 1998). The *n* was the measured sunshine hours and the data for *n* was obtained from near the weather observatory, and N was the maximum possible sunshine hours.

The fraction of intercepted radiation (*Fi*) of maize and soybean in sole and intercropping systems was calculated using the exponential equation from their respective LAI values (Monteith and Elston, 1983).


$$F_{i}=1-exp\;\left(-k\;\times LAI\right)$$

Where, k was the extinction coefficient for total solar radiation (Monteith, 1977; Muurinen and Peltonen-Sainio, 2006), and the values of k for maize and soybean were 0.70 (Lindquist et al., 2005) and 0.45 (Zhang et al., 2014), respectively.

The total amount of incident photosynthetically active radiation (*Si*) was determined by multiplying the total incident radiation by 0.50 because researchers have concluded that the incident photosynthetically active radiation is equal to half (50%) of the daily total incident radiation (Szeicz, 1974; Sinclair and Muchow, 1999; Tesfaye et al., 2006). Then, the amount of intercepted radiation (*Sa*) for maize and soybean under sole and intercropping systems was calculated using the following equation (Szeicz, 1974).


$$Sa=F_{i}\times S_{i}$$

Finally, the radiation use efficiency (RUE) of maize and soybean under sole and intercropping systems were calculated individually using the following equation (Monteith, 1977).


$$RUE=\frac{TDM}{\sum Sa}$$

Where, *TDM* was the total dry matter of maize or soybean, *∑Sa* was the cumulative intercepted photosynthetically active radiation of maize or soybean.

For calculating water use efficiency (WUE), we first measured the total water use (TWU) of maize and soybean in different treatments using the simplified water balance equation (Raza et al., 2021b).


TWU=P+IW+SWs−SWh

Where *P* was the total precipitation (mm) received during the whole growing period (from February to July), *IW* was the total amount of applied irrigation water (mm), *SWs* and *SWh* were the soil water content (mm) at sowing and harvesting of the experiment, respectively. Then, the water use efficiency of both crops was calculated using the following equation (Zhang et al., 1998):


WUE=GYTWU

Where, *GY* was the grain yield of maize or soybean in intercropping or sole cropping systems, and T*WU* was the total water use calculated using the simplified water balance equation.

Furthermore, we calculated the water equivalent ratio (WER) to estimate the water-use advantage of intercropping over sole cropping system, and the partial WER of maize (WER_Maize_) and soybean (WER_Soybean_), and total WER was calculated using the following equations (Mao et al., 2012):


$$WER_{Maize}=\frac{WUE_{IM}}{WUE_{M}}$$

$$WER_{Soybean}=\frac{WUE_{IS}}{WUE_{S}}$$

$$Total\;WER=WER_{Maize}+\;WER_{Soybean}$$

Where, *WUE_IM_* and *WUE_IS_* were the water use efficiency of intercropped maize and soybean, respectively. The *WUE_M_* and *WUE_S_* were the grain yield of sole cropped maize and soybean, respectively.

We measured the land equivalent ratio (LER) to determine the land use advantage of intercropping over the sole cropping system (Raza et al., 2021b). The partial LER of maize (LER_Maize_) and soybean (LER_Soybean_), and total LER was calculated using the following equations:


$$LER_{Maize}=\frac{GY_{IM}}{GY_{M}}$$

$$LER_{Soybean}=\frac{GY_{IS}}{GY_{S}}$$

$$Total\;LER=LER_{Maize}+\;LER_{Soybean}$$

Where, *GY_IM_* and *GY_IS_* were the grain yield of intercropped maize and soybean, respectively. The *GY_M_* and *GY_S_* were the grain yield of sole cropped maize and soybean, respectively.

### Economic analysis

An economic analysis was performed to assess the economic viability of the maize/soybean intercropping system. Total expenditure for maize and soybean production under intercropping and sole cropping system was included; the cost of land rent, maize and soybean grains, land preparation, fertilizer (i.e., Urea, DAP, and SOP), weeding, thinning, irrigation, harvesting, and threshing of crops. Each treatment’s total income (gross income) was estimated according to the yearly local market prices for maize and soybean grains in Pakistan. The net profit was calculated by subtracting the total expenditure from the total income (Raza et al., 2018).

### Statistical analysis

All data analyses were performed using Statistix 8.1. Significant differences were determined using ANOVA, and the LSD (Least Significance Difference) test was used to compare the means at a 5% probability level. Mean values are presented mean ± SE (standard error), based on the three independent replicates per treatment.

## Results

### Growth parameters

**Figure 3:**
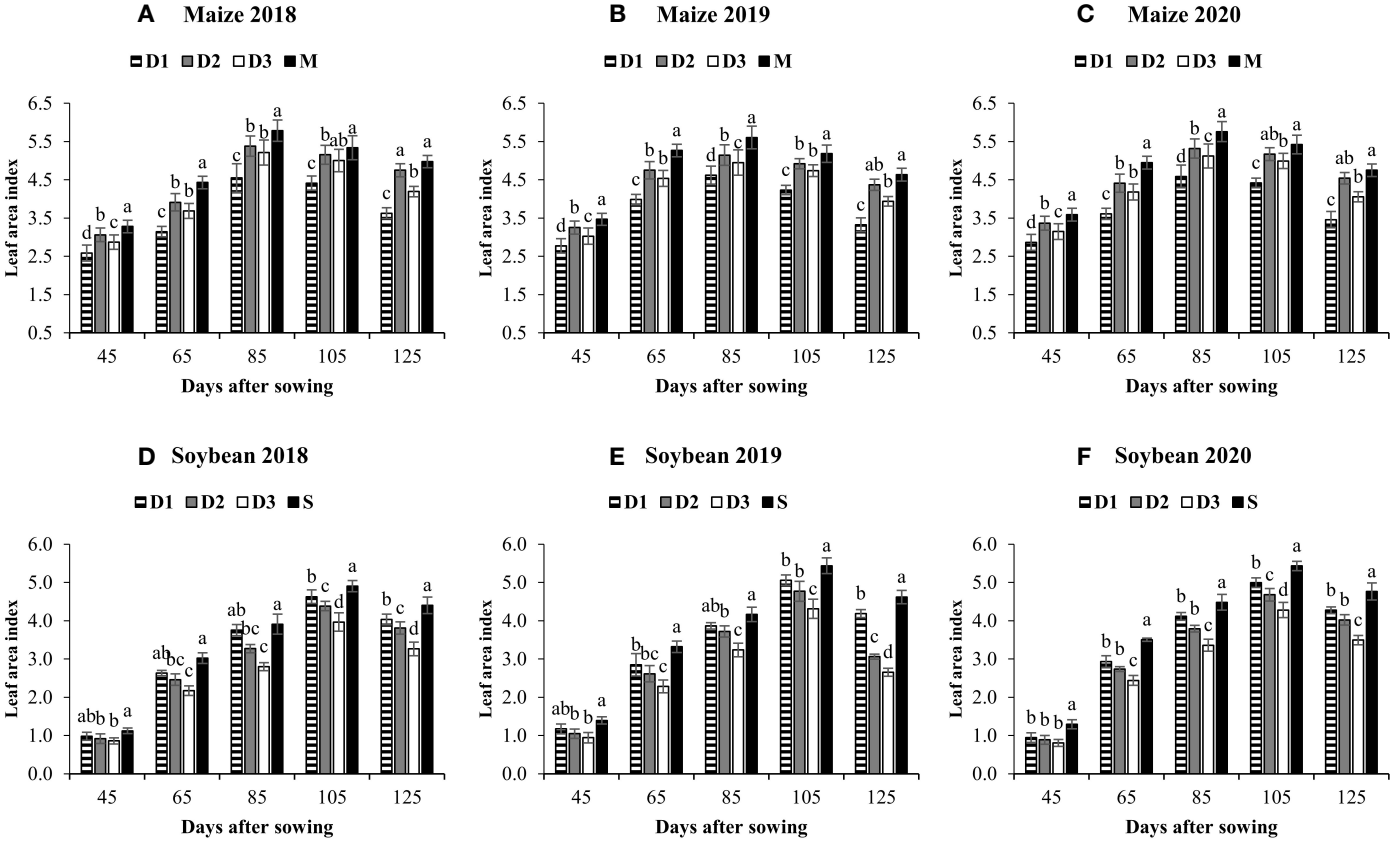
Leaf area index of maize **(A–C)** and soybean **(D–F)** in response to different maize planting densities (6 maize plants m^-2^, low, D_1_; 8 maize plants m^-2^, medium, D_2_; and 10 maize plants m^-2^, high, D_3_) under maize/soybean strip intercropping. Bars show ± standard errors (n = 3). The different lowercase letters within a bar show a significant difference (p < 0.05) among treatments. The M and S represent the sole maize and soybean, respectively.

The LAI of maize and soybean under different planting systems is shown in **Figure 3**. At all sampling times, the LAI of maize and soybean were significantly lower under intercropping than sole maize and soybean. In intercropping treatments, at the final sampling time (125 DAS), the average highest soybean (4.2) and maize (4.6) LAI was measured under D_1_ and D_2_, whereas the average lowest soybean (3.1) and maize (3.5) LAI was recorded in D_3_ and D_1_, respectively. However, at all sampling times, the total LAI of maize and soybean in intercropping treatments was significantly higher than M and S (**Table 3**). For instance, at 125 DAS, the total LAI in D_1_, D_2_, and D_3_,

**Table 3:** Total leaf area index and total dry matter of maize and soybean at 45, 65, 85, 105, and 125 days after sowing (DAS) under different maize/soybean strip intercropping treatments and sole cropping of maize and soybean.

Year	Treatments		Total leaf area index					Total dry matter (g plant^-1^)				
		(Maize leaf area index + soybean leaf area index)						(Maize dry matter + soybean dry matter)				
		45 DAS	65 DAS	85 DAS	105 DAS	125 DAS	45 DAS		65 DAS	85 DAS	105 DAS	125 DAS
**2018**	**D_1_**	3.6 ± 0.2b	5.8 ± 0.2a	8.3 ± 0.4ab	9.0 ± 0.1a	7.7 ± 0.2b	23.9 ± 3.4a		117.8 ± 9.6a	201.6 ± 21.7a	278.0 ± 28.3a	315.1 ± 37.4a
	**D_2_**	4.0 ± 0.2a	6.4 ± 0.1a	8.7 ± 0.2a	9.5 ± 0.2a	8.6 ± 0.3a	23.7 ± 2.6a		122.8 ± 7.4a	203.2 ± 10.1a	300.6 ± 12.8a	345.4 ± 22.8a
	**D_3_**	3.7 ± 0.2b	5.9 ± 0.3a	8.0 ± 0.4b	9.0 ± 0.2a	7.5 ± 0.3b	17.5 ± 1.2b		86.2 ± 3.8b	137.9 ± 6.4b	202.0 ± 18.6b	232.5 ± 20.7b
	**M**	3.3 ± 0.2c	4.4 ± 0.2b	5.8 ± 0.3c	5.3 ± 0.3b	5.0 ± 0.2c	15.7 ± 1.7b		126.7 ± 12.1a	207.1 ± 6.8a	296.0 ± 16.9a	324.2 ± 12.1a
	**S**	1.1 ± 0.1d	3.0 ± 0.1c	3.9 ± 0.3d	4.9 ± 0.1b	4.4 ± 0.2c	15.1 ± 1.3b		24.5 ± 2.2c	45.1 ± 4.6c	56.6 ± 4.3c	65.2 ± 4.8c
	**LSD**	0.3	0.6	0.5	0.6	0.6	5.4		22.8	28.5	37.4	42.8
**2019**	**D_1_**	3.9 ± 0.2a	6.8 ± 0.4a	8.5 ± 0.3ab	9.3 ± 0.1ab	7.5 ± 0.2	30.9 ± 3.2a		151.7 ± 14.2a	235.4 ± 27.7a	325.2 ± 34.2a	375.5 ± 39.7a
	**D_2_**	4.3 ± 0.1a	7.4 ± 0.4a	8.9 ± 0.2a	9.7 ± 0.3ab	7.4 ± 0.2	31.4 ± 3.5a		159.6 ± 11.3a	238.2 ± 21.3a	336.8 ± 23.8a	389.1 ± 29.1a
	**D_3_**	4.0 ± 0.1a	6.8 ± 0.4a	8.2 ± 0.5b	9.1 ± 0.3b	6.6 ± 0.2	23.5 ± 1.3b		113.4 ± 6.5b	172.2 ± 11.2b	236.2 ± 18.8b	270.1 ± 21.2b
	**M**	3.5 ± 0.2b	5.3 ± 0.2b	5.6 ± 0.3c	5.2 ± 0.2c	4.6 ± 0.2	19.5 ± 1.6b		158.7 ± 12.6a	241.6 ± 12.6a	317.8 ± 18.6a	357.6 ± 21.1a
	**S**	1.4 ± 0.1c	3.3 ± 0.2c	4.2 ± 0.2d	5.4 ± 0.2c	4.6 ± 0.2	21.2 ± 1.4b		35.1 ± 1.8c	45.4 ± 6.6c	60.1 ± 6.5c	71.2 ± 3.6c
	**LSD**	0.4	0.8	0.5	0.6	0.5	4.0		25.6	37.1	35.2	46.9
**2020**	**D_1_**	3.8 ± 0.2b	6.5 ± 0.3a	8.7 ± 0.3ab	9.4 ± 0.1ab	7.7 ± 0.2b	26.1 ± 3.1a		137.3 ± 12.8a	212.7 ± 25.0a	301.8 ± 30.1a	348.1 ± 36.3a
	**D_2_**	4.3 ± 0.1a	7.1 ± 0.3a	9.1 ± 0.2a	9.8 ± 0.3a	8.6 ± 0.3a	26.2 ± 2.8a		146.3 ± 10.2a	214.8 ± 16.1a	305.0 ± 18.1a	350.1 ± 25.6a
	**D_3_**	4.0 ± 0.2ab	6.6 ± 0.3a	8.5 ± 0.5b	9.3 ± 0.3b	7.6 ± 0.2b	18.8 ± 1.4b		102.5 ± 6.7b	155.1 ± 8.9b	212.6 ± 12.7b	245.9 ± 15.8b
	**M**	3.6 ± 0.2b	4.9 ± 0.2b	5.8 ± 0.3c	5.4 ± 0.2c	4.8 ± 0.2c	18.4 ± 1.3b		151.7 ± 11.6a	224.3 ± 13.8a	311.4 ± 12.8a	334.3 ± 18.7a
	**S**	1.3 ± 0.1c	3.5 ± 0.0c	4.5 ± 0.2d	5.4 ± 0.1d	4.8 ± 0.2c	16.3 ± 0.4b		26.3 ± 1.9c	41.7 ± 5.2c	54.7 ± 4.9c	57.9 ± 9.5c
	**LSD**	0.4	0.6	0.5	0.6	0.6	4.3		23.2	34.9	37.4	37.3

The D_1_ (6 maize plants m^-2^, low, D_1_), D_2_ (8 maize plants m^-2^, medium, D_2_), and D_3_ (10 maize plants m^-2^, high, D_3_) represent the three maize/soybean intercropping treatments differing with maize plant density. The M refers to the sole cropping of maize, and the S refers to the sole cropping of soybean. Bars show ± standard errors, (n = 3). The lowercase letters within a bar show a significant difference (p< 0.05) among treatments.

**Figure 4:**
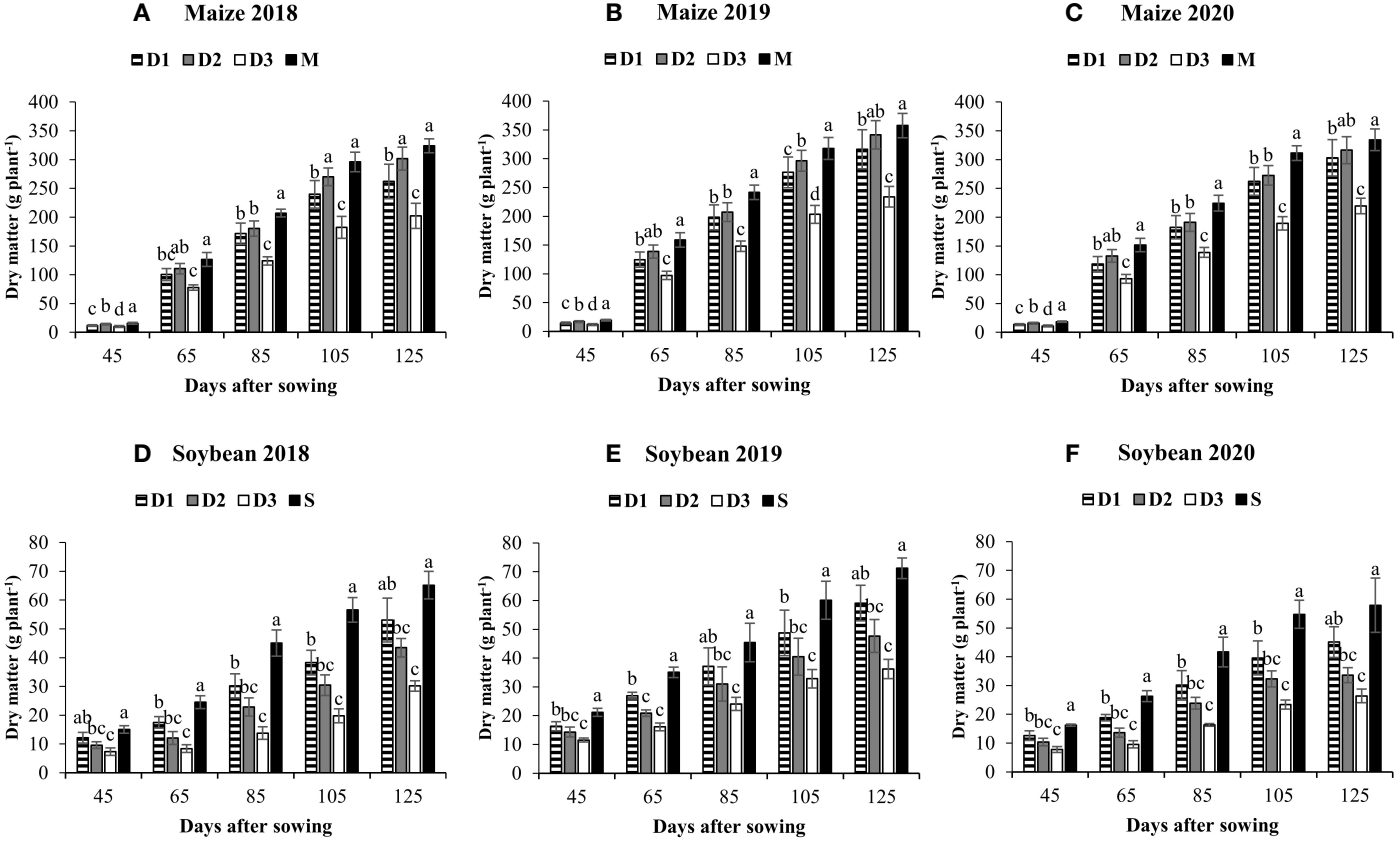
Dry matter of maize **(A–C)** and soybean **(D–F)** in response to different maize planting densities (6 maize plants m^-2^, low, D_1_; 8 maize plants m^-2^, medium, D_2_; and 10 maize plants m^-2^, high, D_3_) under maize/soybean strip intercropping. Bars show ± standard errors (n = 3). The different lowercase letters within a bar show a significant difference (p < 0.05) among treatments. The M and S represent the sole maize and soybean, respectively.

Different treatments significantly affected the total dry matter production of maize and soybean. Across different sampling stages and treatments, maize and soybean plants accumulated higher dry matter in M and S, respectively, than intercropping treatments. In contrast, at the final sampling stage (125 DAS), the average total dry matter (maize dry matter + soybean dry matter; **Table 3**) of D_2_ (361.2 g plant^-1^) was higher than the corresponding values of dry matter in M (338.7 g plant^-1^) and S (64.8 g plant^-1^). In intercropping treatments, maize accumulated the highest (319.9 g plant^-1^) and lowest (218.6 g plant^-1^) dry matter under D_2_ and D_3_, while soybean accumulated the maximum (52.4 g plant^-1^) and minimum (30.9 g plant^-1^) dry matter in D_1_ and D_3_, respectively (**Figure 4**). In addition, different maize planting density treatments in intercropping not only affected dry matter production of intercrops but also changed dry matter partitioning in various plant parts of maize (**Table 4**) and soybean (**Table 5**). For example, across the years, at 125 DAS, treatment D_2_ significantly increased dry matter of maize grains by 13% and 46% compared to D_1_ and D_3_, while treatment D_1_ enhanced dry matter of soybean grains by 21% and 47% compared to D_2_ and D_3_, respectively. Whereas, relative to D_2_, the treatment D_3_ significantly decreased dry matter of maize and soybean roots (by 29% and 19%), straw (by 32% and 29%), and grains (by 31% and 18%), respectively, indicating that the high maize planting density in intercropping caused a significant reduction in dry matter accumulation and partitioning to economic parts (i. e., grains).

**Table 4:** Dry matter partitioning in different plant parts of maize at 45, 65, 85, 105, and 125 days after sowing (DAS) under different maize/soybean strip intercropping treatments and sole cropping of maize.

Year	Treatments	Maize dry matter distribution (g plant^-1^)											
		45 DAS		65 DAS		85 DAS		105 DAS			125 DAS		
		Root	Straw	Root	Straw	Root	Straw	Root	Straw	Grain	Root	Straw	Grain
**2018**	**D_1_**	1.4 ± 0.1b	10.3 ± 1.5c	11.7 ± 2.1b	88.6 ± 8.6ab	15.9 ± 2.2a	155.6 ± 16.0b	18.3 ± 2.1b	177.4 ± 17.4	44.0 ± 5.4bc	21.4 ± 3.1b	169.1 ± 17.4b	71.5 ± 9.9c
	**D_2_**	1.7 ± 0.2a	12.4 ± 1.4b	14.2 ± 1.5a	96.5 ± 7.8a	17.3 ± 1.7a	163.0 ± 11.7ab	19.3 ± 1.7b	195.1 ± 11.2	55.8 ± 6.7ab	23.6 ± 2.3b	188.9 ± 11.2ab	89.5 ± 9.7b
	**D_3_**	1.1 ± 0.2c	9.0 ± 1.3d	9.4 ± 1.0c	68.4 ± 3.8b	12.0 ± 1.0b	112.2 ± 6.1c	13.4 ± 0.9c	132.4 ± 13.1	36.4 ± 6.2c	16.4 ± 1.5c	127.4 ± 13.1c	58.5 ± 8.2d
	**M**	1.8 ± 0.1a	13.9 ± 1.6a	16.0 ± 1.5a	110.6 ± 11.8a	19.3 ± 2.6a	187.8 ± 7.8a	21.4 ± 1.9a	206.1 ± 10.4	68.6 ± 9.1a	26.3 ± 2.7a	199.9 ± 10.4a	98.0 ± 8.0a
	**LSD**	0.25	0.98	2.20	24.91	3.39	25.39	1.95	28.02	13.61	2.56	28.05	5.51
**2019**	**D_1_**	2.1 ± 0.2c	12.5 ± 1.5c	13.9 ± 2.0c	110.8 ± 11.4bc	19.5 ± 2.0b	178.7 ± 19.5b	22.4 ± 3.1b	201.8 ± 19.3b	52.2 ± 4.4b	26.2 ± 3.6b	193.4 ± 19.3b	96.8 ± 11.1b
	**D_2_**	2.3 ± 0.2b	14.7 ± 1.6b	16.6 ± 1.7b	122.2 ± 9.6ab	20.4 ± 1.5b	186.8 ± 14.8b	24.2 ± 1.7ab	212.4 ± 14.3ab	59.7 ± 5.4ab	28.2 ± 2.6b	206.2 ± 14.3ab	107.0 ± 8.5a
	**D_3_**	1.6 ± 0.2d	10.3 ± 1.5d	11.0 ± 1.4d	86.3 ± 6.0c	14.6 ± 1.0c	133.6 ± 8.1c	17.0 ± 1.4c	145.1 ± 10.2c	41.2 ± 4.8c	20.0 ± 2.3c	140.1 ± 10.2c	73.7 ± 5.7c
	**M**	2.5 ± 0.2a	17.0 ± 1.4a	19.1 ± 1.6a	139.7 ± 11.9a	23.0 ± 1.4a	218.5 ± 12.0a	26.7 ± 1.5a	221.9 ± 10.9a	69.3 ± 6.9a	31.8 ± 2.8a	217.3 ± 10.1a	108.4 ± 8.9a
	**LSD**	0.20	1.36	2.24	25.65	1.66	31.20	3.02	17.74	9.81	2.04	18.41	7.64
**2020**	**D_1_**	2.1 ± 0.2b	11.4 ± 1.3c	12.9 ± 1.7c	105.5 ± 11.4b	18.5 ± 1.6	164.1 ± 18.7b	21.1 ± 3.2b	190.9 ± 17.5b	50.1 ± 3.7b	24.3 ± 3.4b	182.6 ± 17.5b	96.1 ± 10.7a
	**D_2_**	2.2 ± 0.2b	13.5 ± 1.4b	15.4 ± 1.6b	117.4 ± 9.3ab	19.0 ± 1.3	172.0 ± 14.3b	23.2 ± 1.5ab	195.4 ± 13.9b	54.1 ± 3.9ab	26.2 ± 2.4ab	189.1 ± 13.9ab	101.2 ± 7.8a
	**D_3_**	1.7 ± 0.2c	9.3 ± 1.4d	10.2 ± 1.4d	82.7 ± 6.3c	13.7 ± 0.8	125.1 ± 7.9c	16.3 ± 1.4c	134.3 ± 7.3c	38.6 ± 3.5c	18.7 ± 2.3c	129.3 ± 7.3c	71.5 ± 4.2b
	**M**	2.5 ± 0.2a	15.9 ± 1.1a	17.8 ± 1.4a	133.8 ± 10.8a	21.6 ± 1.5	202.7 ± 12.4a	25.5 ± 1.1a	225.1 ± 10.3a	60.8 ± 4.8a	28.7 ± 3.2a	201.2 ± 13.1a	104.5 ± 3.2a
	**LSD**	0.21	1.53	2.16	22.67	2.65	30.16	3.53	29.12	7.16	2.49	18.14	14.05

The D_1_ (6 maize plants m^-2^, low, D_1_), D_2_ (8 maize plants m^-2^, medium, D_2_), and D_3_ (10 maize plants m^-2^, high, D_3_) represent the three maize/soybean intercropping treatments differing with maize plant density. The M refers to the sole cropping of maize. Bars show ± standard errors, (n = 3). The lowercase letters within a bar show a significant difference (p< 0.05) among treatments.

**Table 5:** Dry matter partitioning in different plant parts of soybean at 45, 65, 85, 105, and 125 days after sowing (DAS) under different maize/soybean strip intercropping treatments and sole cropping of soybean.

Year	Treatments	Soybean dry matter distribution (g plant^-1^)											
		45 DAS		65 DAS		85 DAS		105 DAS			125 DAS		
		Root	Straw	Root	Straw	Root	Straw	Root	Straw	Grain	Root	Straw	Grain
**2018**	**D_1_**	0.9 ± 0.1ab	11.3 ± 1.8ab	1.7 ± 0.2ab	15.8 ± 2.0b	4.1 ± 0.5b	26.1 ± 3.9ab	4.7 ± 0.5b	29.2 ± 2.7b	4.3 ± 1.3b	8.5 ± 0.6b	37.1 ± 6.2ab	7.5 ± 0.9b
	**D_2_**	0.8 ± 0.1b	8.8 ± 1.1bc	1.4 ± 0.1bc	10.7 ± 2.2bc	3.4 ± 0.3bc	19.4 ± 3.4bc	3.9 ± 0.5c	22.9 ± 4.5b	3.7 ± 0.6bc	6.7 ± 0.2bc	30.4 ± 2.8bc	6.3 ± 0.7bc
	**D_3_**	0.7 ± 0.1b	6.6 ± 1.4c	1.3 ± 0.2c	7.1 ± 1.5c	2.9 ± 0.3c	10.9 ± 1.9c	3.3 ± 0.4c	14.1 ± 2.5bc	2.4 ± 0.7c	5.2 ± 0.8c	19.8 ± 1.7c	5.1 ± 0.7c
	**S**	1.0 ± 0.2a	14.1 ± 1.4a	2.1 ± 0.1a	22.5 ± 2.1a	5.3 ± 0.5a	39.7 ± 4.5a	5.6 ± 0.7a	43.3 ± 3.0a	7.6 ± 1.6a	11.9 ± 0.6a	43.6 ± 3.3a	9.7 ± 1.2a
	**LSD**	0.19	4.53	0.38	6.51	1.00	13.99	0.62	12.79	1.93	2.17	11.55	2.01
**2019**	**D_1_**	1.2 ± 0.1ab	15.1 ± 1.5b	2.5 ± 0.4a	24.5 ± 1.0b	4.5 ± 0.7b	32.7 ± 5.8ab	5.7 ± 0.9ab	37.5 ± 6.2ab	5.6 ± 0.9b	9.2 ± 1.8ab	41.3 ± 4.2ab	8.6 ± 0.4b
	**D_2_**	1.1 ± 0.1b	13.3 ± 1.7bc	1.9 ± 0.2b	18.9 ± 1.1c	3.9 ± 0.8bc	27.1 ± 5.3bc	4.4 ± 0.9bc	31.2 ± 4.8bc	4.9 ± 1.0bc	7.7 ± 1.7bc	32.9 ± 4.0bc	7.1 ± 0.3c
	**D_3_**	0.9 ± 0.1b	10.7 ± 0.7c	1.4 ± 0.2c	14.7 ± 1.4c	3.2 ± 0.3c	20.9 ± 2.1c	3.9 ± 0.3c	25.2 ± 2.7c	3.7 ± 0.2c	6.4 ± 0.9c	23.8 ± 2.0c	6.0 ± 0.5c
	**S**	1.6 ± 0.2a	19.5 ± 1.2a	2.8 ± 0.3a	32.3 ± 1.6a	6.1 ± 0.9a	39.3 ± 5.8a	6.9 ± 0.6a	46.0 ± 5.0a	7.3 ± 1.1a	10.7 ± 1.4a	50.3 ± 1.7a	10.1 ± 0.8a
	**LSD**	0.46	3.71	0.36	5.17	0.96	8.35	1.39	8.99	1.57	2.52	9.23	1.17
**2020**	**D_1_**	1.0 ± 0.1ab	11.6 ± 1.6ab	2.0 ± 0.3a	16.8 ± 1.1b	4.1 ± 0.5b	26.1 ± 4.5ab	5.0 ± 0.7b	30.0 ± 4.3b	4.6 ± 1.2b	6.8 ± 1.3ab	31.8 ± 3.8ab	6.6 ± 0.5b
	**D_2_**	0.9 ± 0.1b	9.5 ± 1.3bc	1.6 ± 0.2b	12.0 ± 1.6bc	3.5 ± 0.5bc	20.3 ± 1.9bc	4.0 ± 0.6c	24.3 ± 2.7bc	4.1 ± 0.7bc	5.1 ± 1.0bc	23.2 ± 1.6bc	5.4 ± 0.6bc
	**D_3_**	0.8 ± 0.1b	7.1 ± 1.1c	1.3 ± 0.2b	8.3 ± 1.4c	2.9 ± 0.2c	13.4 ± 0.2c	3.4 ± 0.3c	17.0 ± 1.0c	2.9 ± 0.4c	4.1 ± 0.9c	17.9 ± 1.4c	4.3 ± 0.5c
	**S**	1.2 ± 0.2a	15.0 ± 0.3a	2.3 ± 0.2a	24.0 ± 1.8a	5.3 ± 0.5a	36.4 ± 4.7a	6.0 ± 0.5a	41.5 ± 3.2a	7.2 ± 1.3a	8.9 ± 1.2a	40.5 ± 8.4a	8.5 ± 0.9a
	**LSD**	0.29	3.58	0.32	5.51	0.81	11.01	0.71	10.24	1.64	2.27	12.84	1.32

The D_1_ (6 maize plants m^-2^, low, D_1_), D_2_ (8 maize plants m^-2^, medium, D_2_), and D_3_ (10 maize plants m^-2^, high, D_3_) represent the three maize/soybean intercropping treatments differing with maize plant density. The S refers to the sole cropping of soybean. Bars show ± standard errors, (n = 3). The lowercase letters within a bar show a significant difference (p< 0.05) among treatments.

### Crop level yields and system-level yield

**Figure 5:**
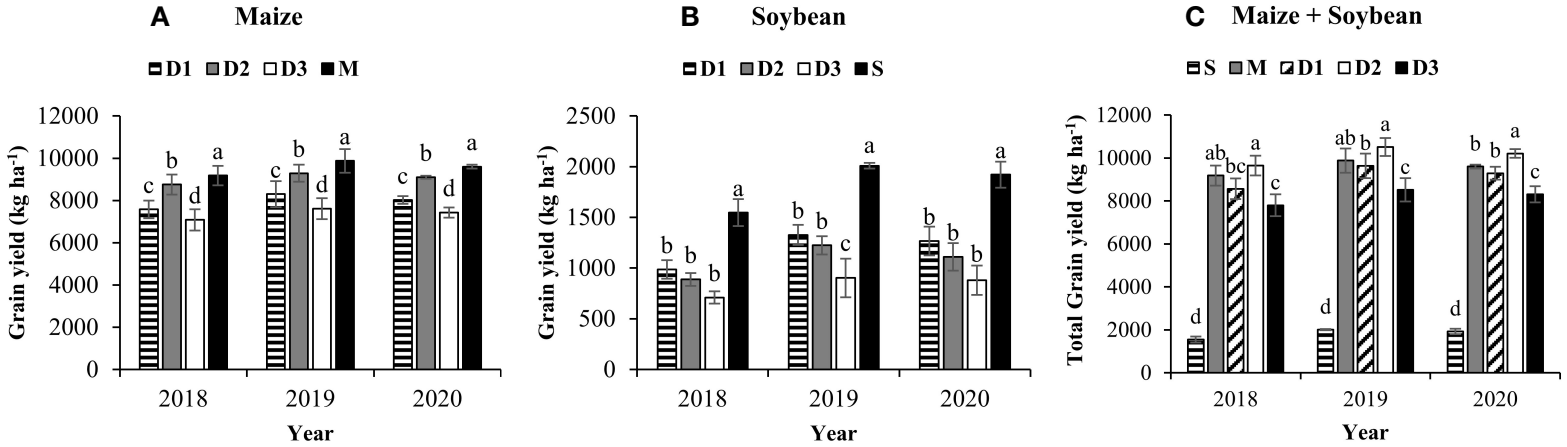
Three years average grain yield of maize **(A)**, soybean **(B)**, total grain yield **(C)** in response to different maize planting densities (6 maize plants m^-2^, low, D_1_; 8 maize plants m^-2^, medium, D_2_; and 10 maize plants m^-2^, high, D_3_) under maize/soybean strip intercropping. Bars show ± standard errors (n = 3). The different lowercase letters within a bar show a significant difference (p < 0.05) among treatments. The M and S represent the sole maize and soybean, respectively.

Grain yield by the intercropped maize and soybean in D_1_, D_2_, and D_3_, compared to sole cropping treatments, is presented in **Figure 5**. The grain yield of maize and soybean in intercropping treatments ranged from 7376.9 to 9047.5 kg ha^-1^ and 830.9 to 1193.5 kg ha^-1^, respectively, which were significantly lower than the three-years average grain yield of M (9553.7 kg ha^-1^) and S (1826.2 kg ha^-1^). However, across the years, the total grain yield of maize and soybean was significantly higher in D_2_ (10122.5 kg ha^-1^) compared to D_1_ (9160.7 kg ha^-1^) and D_3_ (8207.9 kg ha^-1^), and it was also higher than the grain yield of M and S **(** **Figure 5C****)**. Furthermore, among the intercropping treatments, the grain yield of maize significantly increased with increasing maize density from 6 maize plants m^-2^ (D_1_) to 8 maize plants m^-2^ (D_2_), while it decreased under 10 maize plants m^-2^ (D_3_). Contrarily, soybean grain yield significantly reduced with increasing maize density, and the maximum (1193.5 kg ha^-1^) and minimum (830.9 kg ha^-1^) soybean grain yield were obtained in D_1_ and D_3_, respectively. Overall, in D_1_, D_2_, and D_3_, maize produced 83%, 95%, and 77% of M yield, and soybean produced 65%, 59%, and 45% of S yield, respectively.

### Resource (water and radiation) utilization dynamics

The RUE of maize and soybean differed significantly in all treatments, and data are presented in **Table 6**. Across the years, the partial RUE of intercropped maize (3.5 g MJ^-1^ in D_1_, 5.2 g MJ^-1^ in D_2_, and 4.3 g MJ^-1^ in D_3_) and soybean (2.5 g MJ^-1^ in D_1_, 2.1 g MJ^-1^ in D_2_, and 1.8 g MJ^-1^ in D_3_) were significantly lower than the corresponding values of M (5.9 g MJ^-1^) and S (3.2 g MJ^-1^). However, the total RUE of maize and soybean in intercropping was considerably higher than that of the M and S, indicating the advantage of intercropping in utilizing the sunlight than sole systems. Additionally, in intercropping, the RUE of maize was higher than that of soybean, demonstrating the dominance of maize over soybean. On average, D_2_ enhanced the total RUE by 20% and 18% compared to D_1_ and D_3_, respectively.

**Table 6:** Radiation-use-efficiency (RUE), water-use-efficiency (WUE), water equivalent ratio (WER), and land equivalent ratio (LER) of maize and soybean under different maize/soybean strip intercropping treatments and sole cropping of maize and soybean.

Year	Treatments	Radiation use efficiency (g MJ^-1^)			Water use efficiency (kg ha^-1^ mm^-1^)			Water equivalent ratio			Land equivalent ratio		
		Partial RUE		Total RUE	Partial WUE		Total WUE	Partial WER		Total WER	Partial LER		Total LER
		mRUE	sRUE	mRUE + sRUE	mWUE	sWUE	mWUE + sWUE	mWER	sWER	mWER + sWER	mLER	sLER	mLER + sLER
**2018**	**D_1_**	3.2 ± 0.49d	2.5 ± 0.38b	5.6 ± 0.41b	14.9 ± 1.0b	1.9 ± 0.2b	16.8 ± 1.0b	0.84 ± 0.02b	0.65 ± 0.11^NS^	1.50 ± 0.13a	0.83 ± 0.01b	0.65 ± 0.11a	1.48 ± 0.11b
	**D_2_**	4.9 ± 0.37b	2.2 ± 0.23bc	7.0 ± 0.24a	16.8 ± 1.6a	1.7 ± 0.2b	18.5 ± 0.7a	0.95 ± 0.01a	0.58 ± 0.04	1.53 ± 0.05a	0.95 ± 0.01a	0.58 ± 0.06ab	1.53 ± 0.06a
	**D_3_**	4.0 ± 0.50c	1.7 ± 0.39c	5.7 ± 0.09b	13.9 ± 1.3b	1.4 ± 0.5b	15.2 ± 1.1c	0.79 ± 0.04b	0.47 ± 0.04	1.25 ± 0.09b	0.77 ± 0.03c	0.46 ± 0.04b	1.23 ± 0.07c
	**M**	5.7 ± 0.87a	–	–	17.6 ± 0.7a	–	–	–	–	–	–	–	–
	**S**	–	3.3 ± 0.50a	–	–	2.9 ± 0.3a	–	–	–	–	–	–	–
	**LSD**	0.05	0.69	0.83	1.12	0.50	1.42	0.08	–	0.18	0.049	0.13	0.13
**2019**	**D_1_**	3.8 ± 0.41d	2.8 ± 0.34b	6.6 ± 0.33b	15.8 ± 1.1c	2.5 ± 0.3b	18.3 ± 1.0b	0.83 ± 0.02b	0.66 ± 0.05a	1.49 ± 0.05a	0.84 ± 0.02b	0.66 ± 0.06a	1.50 ± 0.06a
	**D_2_**	5.5 ± 0.35b	2.4 ± 0.37bc	7.9 ± 0.38a	17.8 ± 1.6b	2.3 ± 0.5b	20.1 ± 0.8a	0.94 ± 0.01a	0.61 ± 0.04a	1.55 ± 0.03a	0.94 ± 0.01a	0.61 ± 0.05a	1.55 ± 0.05a
	**D_3_**	4.7 ± 0.44c	2.1 ± 0.35c	6.7 ± 0.18b	14.6 ± 1.4d	1.7 ± 0.6c	16.3 ± 1.2c	0.77 ± 0.02b	0.45 ± 0.10b	1.22 ± 0.11b	0.77 ± 0.01c	0.45 ± 0.10b	1.22 ± 0.11b
	**M**	6.3 ± 0.87a	–	–	18.9 ± 1.7a	–	–	–	–	–	–	–	–
	**S**	–	3.6 ± 0.44a	–	–	3.8 ± 0.3a	–	–	–	–	–	–	–
	**LSD**	0.45	0.59	0.53	0.95	0.58	1.61	0.07	0.15	0.18	0.06	0.13	0.14
**2020**	**D_1_**	3.7 ± 0.32d	2.1 ± 0.31b	5.8 ± 0.27b	12.3 ± 0.4c	2.0 ± 0.3b	14.3 ± 0.5b	0.83 ± 0.01b	0.67 ± 0.11a	1.50 ± 0.12a	0.83 ± 0.01b	0.67 ± 0.11a	1.51 ± 0.12a
	**D_2_**	5.1 ± 0.28b	1.7 ± 0.20b	6.8 ± 0.26a	14.0 ± 1.1b	1.7 ± 0.3b	15.8 ± 0.4a	0.95 ± 0.02a	0.59 ± 0.09a	1.54 ± 0.10a	0.95 ± 0.01a	0.59 ± 0.10b	1.53 ± 0.10a
	**D_3_**	4.4 ± 0.40c	1.5 ± 0.20b	5.9 ± 0.13b	11.4 ± 0.9d	1.4 ± 0.5b	12.8 ± 0.5c	0.77 ± 0.01c	0.47 ± 0.09b	1.24 ± 0.10b	0.77 ± 0.02c	0.47 ± 0.09c	1.24 ± 0.11b
	**M**	5.9 ± 0.76a	–	–	14.8 ± 0.8a	–	–	–	–	–	–	–	–
	**S**	–	2.9 ± 0.59a	–	–	3.0 ± 0.3a	–	–	–	–	–	–	–
	**LSD**	0.42	0.65	0.62	0.57	0.64	0.59	0.05	0.10	0.09	0.05	0.07	0.08

The D_1_ (6 maize plants m^-2^, low, D_1_), D_2_ (8 maize plants m^-2^, medium, D_2_), and D_3_ (10 maize plants m^-2^, high, D_3_) represent the three maize/soybean intercropping treatments differing with maize plant density. The M and S refers to the sole cropping of maize and soybean, respectively. Bars show ± standard errors, (n = 3). The lowercase letters within a bar show a significant difference (p< 0.05) among treatments. NS refers to non-significant difference (p< 0.05) among treatments.

There were significant differences in WUE of maize and soybean in intercropping and sole cropping treatments, and data are shown in **Table 6**. Based on average WUE values in three years, the WUE of maize (14.3 kg ha^-1^ mm^-1^ in D_1_, 16.2 kg ha^-1^ mm^-1^ in D_2_, and 13.3 kg ha^-1^ mm^-1^ in D_3_) and soybean (2.1 kg ha^-1^ mm^-1^ in D_1_, 1.9 kg ha^-1^ mm^-1^ in D_2_, and 1.5 kg ha^-1^ mm^-1^ in D_3_) in intercropping treatments was found significantly lower than that of M (17.1 kg ha^-1^ mm^-1^) and S (3.2 kg ha^-1^ mm^-1^), respectively. However, the effect of intercropping on WUE was determined using the values of WER because it characterizes whether the total yield of maize and soybean in D_1_, D_2_, and D_3_ will be produced with more water (WER > 1) or less water (WER< 1) in sole maize and soybean treatments, and data are shown in **Table 6**. In this study, the mean total WER (WER_Maize_ + WER_Soybean_) values of D_1_ (1.50), D_2_ (1.54), and D_3_ (1.24) were consistently higher than unity, demonstrating the water use advantage of intercropping over sole cropping. Moreover, in intercropping treatments, the partial WER values of maize were consistently higher than the partial WER values of soybean, showing that the maize had a competitive advantage over soybean in using the available water. The maximum WER_Maize_ and WER_Soybean_ were in D_2_ and D_1_, while the minimum WER_Maize_ and WER_Soybean_ were in D_1_ and D_3_, respectively.

### Land productivity and economic viability

The total LER (LER_Maize_ + LER_Soybean_) of intercropping treatments ranged from 1.22 to 1.55 in the three years of this experiment, and data are given in **Table 6**. Thus, there was a substantial land-use advantage under intercropping over sole cropping treatments. On average, in intercropping, the total LER was consistently higher in D_2_ (1.54) than D_1_ (1.50) and D_3_ (1.23). Across years and intercropped species, the partial LER values of maize and soybean in intercropping treatments ranged from 0.77 to 0.95 and 0.45 to 0.67, respectively. In intercropping treatments, soybean had the lowest partial LER values, and it decreased with increasing maize planting density. In contrast, maize had the high partial LER values, and it increased from low to medium maize planting density, and then decreased with high maize planting density. Despite the low soybean partial LER values, all the intercropping treatments achieved the high total LER values because the considerable yield of soybean compensated the slight yield loss of maize in D_1_, D_2_, and D_3_ compared to M. Overall, the medium (D_2_) maize planting density treatment increased the total LER by 3% and 25% relative to low (D_1_) and high (D_3_) maize planting density treatments, respectively.

Variations in grain yield directly affected the gross income and net income of D_1_, D_2_, D_3_, M, and S, and data are presented in **Table 7**. Across the years, the highest gross (2624 US $ ha^-1^) and net (1300 US $ ha^-1^) income were obtained under treatment D_2_, whereas the lowest gross (1539 US $ ha^-1^) and net (703 US $ ha^-1^) income were noticed in S treatment. Overall, the intercropping treatment D_2_, enhanced the net income by 63% compared to M and by 85% compared to S, respectively, indicating that the intercropping had an advantage over M and S in utilizing the available resources, i. e., radiation, water, and land.

**Table 7:** Total expenditure and total net income of maize and soybean under different maize/soybean strip intercropping treatments and sole cropping of maize and soybean.

Treatments	Total Expenditure (US $ ha^-1^)			Total Net Income (US $ ha^-1^)			Average (US $ ha^-1^)	
	2018	2019	2020	2018	2019	2020	Expenditure	Net Income
**D_1_**	1542	1220	1131	896	1464	1185	1298	1182
**D_2_**	1574	1246	1155	1051	1574	1274	1325	1300
**D_3_**	1606	1272	1178	511	969	788	1352	756
**M**	1415	1120	1038	623	962	810	1191	798
**S**	993	786	728	559	868	683	836	703

The D_1_ (6 maize plants m^-2^, low, D_1_), D_2_ (8 maize plants m^-2^, medium, D_2_), and D_3_ (10 maize plants m^-2^, high, D_3_) represent the three maize/soybean intercropping treatments differing with maize plant density. The M refers to the sole cropping of maize, and the S refers to the sole cropping of soybean.

## Discussion

The combination of maize and soybean as intercropping is a better option for irrigated areas under semi-arid conditions. Our three-year field study proved this, where we recorded high land- and water-equivalent ratios, showing a substantial increase in land and water use in intercropping treatments over sole cropping systems. Notably, just 50% of the total land was available for maize or soybean in intercropping treatments, while maize or soybean yield in intercropping treatments was higher than half of the sole maize or soybean yield. These results are aligned with the previously observed growth and yield pattern of cereals and legumes under intercropping systems (Li et al., 2020; Raza et al., 2021a). Overall, this shows that the extra yield produced by soybean in intercropping had minor consequences for maize production, and the interaction between maize and soybean was not highly competitive in intercropping treatments. Therefore, the system as a whole (maize + soybean) enhanced the total resource capturing and utilization beyond that of the sole cropping systems due to the complementary resource use of both species in intercropping (Yang et al., 2017; Iqbal et al., 2018; Liu et al., 2018; Ren et al., 2019; Li et al., 2020).

In intercropping, the better growth (measured as leaf area index and total dry matter production) of maize was likely associated with greater light use efficiency (Liu et al., 2018), water use efficiency (Rahman et al., 2017), nutrient accumulation (Ahmed et al., 2018), and plasticity of edge-row plants (Zhu et al., 2016). In contrast, the intercropped soybean growth was significantly lower in intercropping treatments than in sole soybean and this difference was increased with increasing maize density where soybean suffered from heavy maize shading (Yang et al., 2017) and water stress than sole soybean (Raza et al., 2021a). Thus, optimum maize planting density in intercropping (8 maize plants m^-2^) can increase maize yield with maintained soybean yield by improving the light transmittance at the soybean canopy and reducing the intra-specific competition for available resources, especially for light and water (Zhang 2007; Yang et al., 2015; Feng et al., 2020). Additionally, under semi-arid conditions, maize and soybean growth and yield are easily subjected to water stress (Cui et al., 2020). Therefore, the intercropping of maize with soybean could play a vital role in saving water, especially under semi-arid conditions, because intercropping systems reduce water evaporation due to greater canopy closure, which means that intercrops can produce more grains per mm of water than sole crops (Cooper et al., 1987; Wallace, 2000; Raza et al., 2021b).

Compared to past studies (Gao et al., 2010), the enhanced radiation use efficiency in different maize planting density treatments under maize/soybean intercropping was mainly associated with density and planting arrangement advantage. In this study, we planted both crops using the narrow-wide-row planting arrangement (narrow inter-row distance between maize or soybean rows and wide intra-row distance between maize and soybean strips), which gives the edge row advantage and spatial light distribution advantage. Besides, the total planting density (maize planting density + soybean planting density; **Table 1**) in intercropping treatments was considerably higher than sole crops (Feng et al., 2019), which resulted in increased radiation use efficiency as it was followed by a high leaf area index (Raza et al., 2021a). Although the individual leaf area index values of intercrop species were lower in intercropping, but the total leaf area index of maize and soybean was relatively higher than sole crops. This might have resulted in an increased light interception in intercropping, which consequently increased the total radiation use efficiency of maize/soybean intercropping than sole maize or sole soybean. Our results are in line with the previous report (Feng et al., 2019), in which they reported greater light interception and radiation use efficiency in maize/soybean intercropping and linked it with an improved leaf area index, light interception, and dry matter production (Liu et al., 2018). However, the partial RUE of intercropped maize or soybean in intercropping was significantly lower than that of sole maize or soybean, indicating the competition for solar radiations between intercrops in intercropping, as reported in many previous studies (Gao et al., 2010; Feng et al., 2020; Raza et al., 2020). Therefore, the radiation use efficiency of intercropping systems can be increased by selecting the optimum planting density of intercrop species, especially of tall crops (i. e., maize, millet, sorghum, etc.) because it directly influences the light environment of short stature crops (i. e., soybean, peanut, pea, etc.) in cereal legume intercropping systems.

The data of water equivalent ratio indicated that maize/soybean intercropping considerably increased the water use efficiency. Considering that the intercropping had a 175% planting density in D_1_, 200% planting density in D_2_, and 225% planting density in D_3_, indicating that the intercropped soybean and maize produced more seeds mm^-1^ of water than sole maize or soybean because under intercropping treatments the total available water was halved for soybean and maize. In addition, the different maize planting density treatments significantly affected the water use efficiency of intercropped species. The increasing maize density from 8 to 10 maize plants m^-2^ decreased the water use efficiency and partial water equivalent ratio of maize and soybean, suggesting the competition for water first among maize plants and second between maize and soybean plants, which means that appropriate planting density of intercrop species is critical in achieving high water productivity through resource complementarity (Mao et al., 2012), especially under the scenario of limited water resources (Ren et al., 2016). Interestingly, in all treatments, maize produced more grains mm^-1^ of water than soybean because maize had a competitive advantage over soybean in root growth and development, which ultimately increased the water uptake and used in maize than soybean (Raza et al., 2021b). However, despite this asymmetry in water uptake and use between soybean and maize, all intercropping treatments were still advantageous in translating water into grains, as indicated by total grain yields. This improvement in water use efficiency in D_1_, D_2_, and D_3_ might be caused by: (i) the water use efficiency of maize and soybean in intercropping depends on the selection of appropriate planting density, especially of maize (Ren et al., 2016); (ii) medium planting density of maize (8 maize plants m^-2^; D_2_) increased the water use efficiency of maize and maintained the water use efficiency of soybean under maize/soybean intercropping, which in return increased the total water equivalent ratio (Raza et al., 2021b); and (iii) all intercropping treatments were irrigated with the same amount of water as sole soybean or maize but produced more grains mm^-1^ of water, which might be associated with reduced evapotranspiration from the soil and plant surface due to greater canopy closure in intercropping (Cooper et al., 1987; Wallace, 2000). Another possible reason for high WER is related to complementarity in water uptake lower and upper soil depths by maize and soybean, respectively (Bai et al., 2016). However, more research is needed to understand complementarity in water acquisition from different soil depths by intercrops.

Total economic return (net profit) is the main factor for adopting any new planting method or practice (Piepho, 1998; Raza et al., 2019). Agreeing with previous results (Du et al., 2017; Li et al., 2020), the findings of this study demonstrate high resource (radiation, water, and land) use advantages, crop yield stability, and total net profit of all intercropping treatments over the sole maize and sole soybean under semi-arid conditions with irrigation. Additionally, the higher net profit of intercropping over sole cropping suggested that farmers could plant soybean and maize together in intercropping with a minimal overall yield penalty. The improvement in greater economic returns mainly attributed to an extra yield of soybean with maintained maize yield, especially under D_2_, which ultimately increased the total profit by 63% and 85% over sole maize and soybean because, in the local market, the price of soybean is three times expensive than maize price. Therefore, we can conclude that intercropping of soybean with maize, especially at eight maize plants m^-2^, is the better planting practice to obtain high economic returns with limited resources. Moreover, with appropriate planting configuration and density in maize/soybean strip intercropping, farmers can increase soybean production without decreasing the maize production and area, ultimately improving soil fertility and productivity through nitrogen fixation and release of root exudates (Chen et al., 2017). However, future studies are needed to quantify the resource use mechanism of intercropped maize and soybean in intercropping, especially under the changing climate scenarios. For instance, crops under intercropping may perform differently under low light regions (i. e., Sichuan in China), and farmers need to reduce the overall planting density to avoid the mutual shading effect on intercrops.

## Conclusion

The system yield (maize yield + soybean yield), resource utilization (radiation and water), and net income advantages of intercropping over sole cropping were high and consistent over three years, indicating that intercropping is a more effective and profitable planting system than sole systems. Overall, these results indicate that optimizing strip intercropping systems can save 20–50% of water and land, especially under the present scenario of limited resources and climate change. Therefore, we can conclude that intercropping could be a productive and sustainable system to alleviate poverty and drought risk, especially for small landholder farmers in developing countries. However, future studies are required to quantify the resource use mechanism of intercrops in intercropping, particularly in the present climate change scenario. Moreover, intercropping-specific small farm machinery is needed (sowing and haversting specific equipments) to obtain the maximum advantages of intercropping; without resolving this issue, we cannot attain the full benefits of intercropping systems.

## Data availability statement

The original contributions presented in the study are included in the article/supplementary material. Further inquiries can be directed to the corresponding authors.

## Author contributions

MAR, HSY and HG: design and conceived research and writing original draft; RQ, AMD, MHBK, and SH: writing, reviewing and editing; JW, HGT, AS; reviewing, editing and analysis; AM, ERC, AF, and SA: data curation; FY, MS and WY: project administration and supervision; WY: reviewing and supervision. All authors contributed to the article and approved the submitted version.

## Funding

This research was financially supported by the International Cooperation Project of Sichuan Province (2020YFH0126) and the Program on Industrial Technology System of National Soybean (CARS-04-PS19). This publication has been supported by the project OP VVV CZ.02.2.69/0.0/0.0/18_054/0014642 and the Operational Program Integrated Infrastructure within the project: Demand-driven Research for the Sustainable and Innovative Food, Drive4SIFood 313011V336, co-financed by the European Regional Development Fund.

## Acknowledgments

MAR would sincerely express his gratitude to Prof. Athar Mahboob, who made possible whatever he deemed, especially for strip intercropping’s promotion and demonstrations in Pakistan. Even though he found it difficult to substitute his feelings and respect in words, he would say that every aspect regarding your support, including preliminary discussions and funds, encouragement and compliments, belief and love, finally made him to confidently set goal-oriented priorities; you are an honorable and great teacher who brilliantly tracked the points regarding the strengths and weaknesses of my work and personality.

## Conflict of interest

The authors declare that the research was conducted in the absence of any commercial or financial relationships that could be construed as a potential conflict of interest.

## Publisher’s note

All claims expressed in this article are solely those of the authors and do not necessarily represent those of their affiliated organizations, or those of the publisher, the editors and the reviewers. Any product that may be evaluated in this article, or claim that may be made by its manufacturer, is not guaranteed or endorsed by the publisher.
